# Drug attitude inventory is relevant to LAI treatment persistence in schizophrenia: Preliminary results

**DOI:** 10.1192/j.eurpsy.2021.422

**Published:** 2021-08-13

**Authors:** G. D’Anna, L. Tatini, F. Pietrini, A. Ballerini, V. Ricca

**Affiliations:** 1 Department Of Health Sciences, University of Florence, Florence, Italy; 2 Department Of Mental Health And Addictions, Central Tuscany NHS Trust, Florence, Italy

**Keywords:** Drug Attitude Inventory, adherence, schizophrénia, LAI

## Abstract

**Introduction:**

Patients’ attitudes and subjective experience are crucial in the management of severe mental illness, but their practical value is overlooked.

**Objectives:**

To identify predictors of future adherence to LAI antipsychotic maintenance treatment of schizophrenia among socio-demographic, clinical, and psychometric characteristics – including Drug Attitude Inventory-10 (DAI-10) and Subjective Well-being under Neuroleptics short form (SWN-K) scores.

**Methods:**

Retrospective baseline data from 53 clinically stable outpatients with schizophrenia switched from oral to LAI therapy were collected. Patients continuing treatment at the time of analysis (n=29) were compared to those who had discontinued it (n=24). Selected variables were further evaluated in survival analyses.

**Results:**

Between-group differences are presented in Table 1 (**: p<0.01; *: p<0.05).

**Table:** 

	Continued treatment	Discontinued treatment	χ^2^ or t
Treatment persistence (months)	63.79±21.01	23.88±25.80	6.21**
Age (years)	39.17±10.11	35.58±13.39	1.11
Male	15 (51.7%)	13 (54.1%)	0.03
Single	20 (69.0%)	15 (62.5%)	0.25
Instruction (years)	13.28±3.31	11.83±3.56	1.53
Employed	20 (69.0%)	7 (29.2%)	8.32**
Illness duration (years)	17.69±10.53	13.42±11.36	1.42
Previous hospitalisations	2.10±1.32	2.67±1.86	-1.29
MADRS	13.59±9.06	14.67±8.99	-0.43
YMRS	5.52±5.57	6.00±9.94	-0.22
p-PANSS	12.17±5.20	14.38±6.13	-1.41
n-PANSS	10.90±5.39	15.63±7.93	-2.48*
g-PANSS	29.38±10.33	33.63±10.26	-1.49
PANSS	52.66±17.57	63.96±20.61	-2.15*
DAI-10	3.86±4.96	-1.13±5.80	3.38**
SWN-K	74.93±23.07	81.00±15.60	-1.09

Cox regression analysis included instruction, employment, hospitalisations, PANSS subscales and DAI-10 scores: a protective role against treatment discontinuation was outlined only for employment (HR 0.16; 95%CI 0.05-0.50) and higher DAI-10 scores (HR 0.85; 95%CI 0.78-0.94). DAI-10 scores delineated distinct adherence trajectories (Figure 1).

**Figure:**
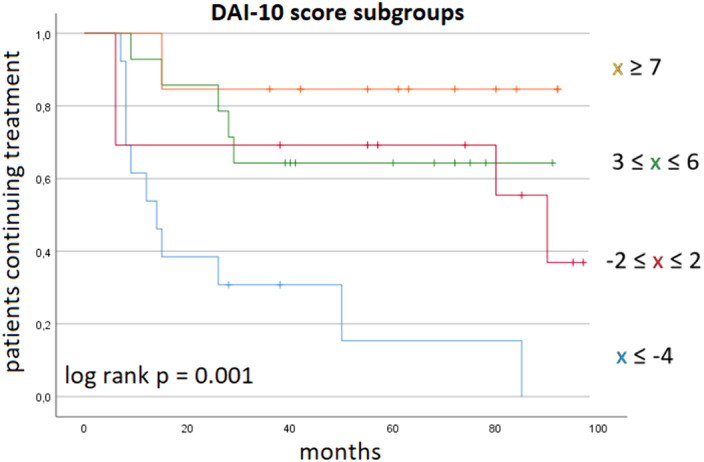

**Conclusions:**

Baseline DAI-10 scores may identify patients at risk of dropout after switching to LAI.

**Disclosure:**

No significant relationships.

